# Caracterización de conjuntivitis infecciosa en un centro de cuarto nivel de atención, Bogotá, Colombia

**DOI:** 10.15446/rsap.V25n6.110422

**Published:** 2023-11-01

**Authors:** Tatiana Urrea-Victoria, Mariana Daza-Betancourt, Hannia Paola Barrios-Bermúdez, María Fernanda Jaimes-Escobar

**Affiliations:** 1 TV: OFT. Esp. Retina y Vitreo. Hospital Universitario San Ignacio. Bogotá, Colombia. turrea@husi.org.co Hospital Universitario San Ignacio Bogotá Colombia turrea@husi.org.co; 2 MD: MD. Hospital Universitario San Ignacio. Bogotá, Colombia. mariana-dazab@javeriana.edu.co Hospital Universitario San Ignacio Bogotá Colombia mariana-dazab@javeriana.edu.co; 3 HB:: MD. Hospital Universitario San Ignacio. Bogotá, Colombia. barrios-h@javeriana.edu.co Hospital Universitario San Ignacio Bogotá Colombia barrios-h@javeriana.edu.co; 4 MJ: MD. Hospital Universitario San Ignacio. Bogotá, Colombia. mariaf.jaimes@javeriana.edu.co Hospital Universitario San Ignacio Bogotá Colombia mariaf.jaimes@javeriana.edu.co

**Keywords:** Covid-19, infección respiratoria por SARS-CoV-2, cuarentena, higiene de manos, medidas de bioseguridad, aislamiento, conjuntivitis infecciosa *(fuente: DeCS*, BIREME), Covid-19, respiratory infection by SARS-CoV-2, quarantine, hand hygiene, biosecurity measures, isolation, infectious conjunctivitis *(source: MeSH*, NLM)

## Abstract

**Objetivo:**

El propósito de este estudio es describir las características clínicas y demográficas de la población que consultó al servicio de urgencias por conjuntivitis desde el 1.o de marzo del 2019 hasta el 1.o de marzo del 2021 y evaluar la frecuencia de presentación de patologías oculares infectocontagiosas, como es el caso de la conjuntivitis bacteriana.

**Métodos:**

Se realizó un estudio descriptivo transversal en una cohorte histórica en el Departamento de Cirugía, Unidad de Oftalmología del Hospital Universitario San Ignacio, Bogotá D.C. desde marzo de 2019 hasta el 1.o de marzo de 2021. El estudio incluyó pacientes con diagnóstico de conjuntivitis bacteriana y pacientes con diagnóstico de conjuntivitis viral sobreinfectada, y excluyó a todos los pacientes con resultado positivo para COVID-19 y a los pacientes con síntomas sugestivos de infección por SARS-CoV-2. De los pacientes, 629 cumplieron con los criterios de selección.

**Resultados:**

Entre marzo del 2019 y abril del 2020 se registraron 534 consultas al servicio de urgencias de la unidad de oftalmología, y a partir del día 24 de abril del 2020 (cuando entró en vigencia la Resolución 666 sobre medidas de bioseguridad) hasta marzo del 2021 se registraron 95 consultas. Gran parte de los pacientes presentaron conjuntivitis viral, seguida por conjuntivitis viral sobreinfectada, y en tercer lugar de conjuntivitis bacteriana.

**Conclusión:**

Se estimó que medidas de bioseguridad como el aislamiento, el lavado frecuente de manos o el uso de tapabocas pudieran explicar la notoria reducción de los casos de propagación de conjuntivitis infecciosa en la comunidad.

## Abstract (en)

**Objective:**

The purpose of this study is to describe the characteristics of the populations that consulted to the emergency service due to conjunctivitis from March 1 of 2019 to March 1 of 2021 and evaluate the frequency of presentation of contagious ocular pathologies like is the case of bacterial conjunctivitis.

**Methods:**

A descriptive cross-sectional study was conducted in a historical cohort in the Department of Surgery, Ophthalmology Unit of the Hospital Universitario San Ignacio, Bogotá D.C. from March 2019 to March 1, 2021. The study included patients with a diagnosis of bacterial conjunctivitis and patients with a diagnosis of superinfected viral conjunctivitis, and excluded all patients with a positive result for COVID-19 and patients with symptoms suggestive of SARS-infectionCoV-2. Of the patients, 629 met the selection criteria.

**Results:**

Between March 2019 and April 2020, 534 consultations were registered in the emergency department of the ophthalmology unit, and from April 24, 2020 (when resolution 666 on biosecurity measures started) until March 2021, 95 consultations were registered. The majority of the patients presented with viral conjunctivitis, followed by superinfected viral conjunctivitis, and bacterial conjunctivitis.

**Conclusion:**

We hypothesize that biosecurity measures such as isolation, frequent hand washing, and the use of face masks could explain the marked reduction in cases of infectious conjunctivitis in the community.

La conjuntivitis es la inflamación unilateral o bilateral de la conjuntiva bulbar y tarsal 1. Puede tener diferentes etiologias, como lo son las causas virales y bacterianas. Ambas son, respectivamente, la primera y la segunda causa de conjuntivitis infecciosas más comunes 2. Entre las causas virales, el 65-90 % de los casos se deben al adenovirus 3. Esta infección, que puede durar de cinco a catorce días, se caracteriza por una secreción acuosa y por presentarse al inicio unilateralmente 4,5. Otros microorganismos que podrían causar la conjuntivitis viral son los enterovirus, coxsackievirus y herpesvirus 5. Los patógenos bacterianos implicados con mayor frecuencia son los estafilococos, en particular *Staphylococcus aureus,* que es el agente causal de más del 50 % de las infecciones bacterianas, seguidos de una gran gama de bacterias gram positivas y gram negativas. La conjuntivitis bacteriana se caracteriza por una secreción amarillenta, verdosa, purulenta, ojo rojo y en ocasiones quemosis 6. Es autolimitada y se puede resolver en 7 a 10 días 3. Los factores de riesgo para tener conjuntivitis son la edad, siendo más prevalente en niños, compartir objetos personales con personas contagiadas por conjuntivitis y el uso de lentes de contacto 6.

Este tipo de infecciones, al ser altamente contagiosas, se pueden transmitir fácilmente a las personas por contacto personal, al toser, estornudar o al tener contacto con superficies u objetos. Por esta razón, es de gran importancia realizar una adecuada higiene de manos con el fin de prevenir la transmisión de esta entidad infectocontagiosa. Las personas que son diagnosticadas con conjuntivitis idealmente deben mantenerse lejos de sus actividades laborales y escolares, para evitar la propagación de esta entidad hasta que el médico lo considere adecuado y se establezca un control infeccioso de la superficie ocular 7.

El objetivo del presente artículo es la caracterización de la población y las frecuencias de las consultas de urgencias oftalmológicas realizadas por los pacientes con signos y síntomas compatibles con conjuntivitis infecciosa, principalmente de etiología bacteriana, en el Hospital Universitario San Ignacio desde marzo del 2019 hasta marzo del 2021.

## MÉTODO

El objetivo del estudio fue describir las características clínicas y demográficas de la población que consulta por conjuntivitis bacteriana e infecciosa en un centro de cuarto nivel durante el periodo de marzo del 2019 hasta marzo del 2021.

En el estudio se incluyeron los pacientes con diagnóstico de conjuntivitis bacteriana y aquellos con diagnóstico de conjuntivitis viral sobreinfectada; y se excluyeron todos los pacientes con resultado positivo para COVID-19 y los que presentaban síntomas sugestivos de infección por SARS-CoV-2. Seiscientos veintinueve pacientes cumplieron con los criterios de selección.

Se llevó a cabo un estudio descriptivo de corte transversal en una cohorte histórica en el Departamento de Cirugía, Unidad de Oftalmología del Hospital Universitario San Ignacio (HUSI), Bogotá D. C., desde marzo del 2019 hasta el 1.o de marzo del 2021. Para el estudio se seleccionaron los pacientes con diagnóstico de conjuntivitis infecciosa atendidos en la Unidad de Oftalmología.

La captura de la información se hizo en la plataforma REDCap, donde se recopilaron datos generales sobre los pacientes (edad, sexo, ocupación, régimen de afiliación) y datos sobre la clínica de la patología (tiempo de evolución de los síntomas, síntomas oculares y asociados, tipo de secreción, clasificación de la conjuntivitis en la historia clínica y comorbilidades de los pacientes). Para la presentación clínica de la patología se consideraron las formas clínicas: conjuntivitis viral, conjuntivitis bacteriana, conjuntivitis viral sobreinfectada y conjuntivitis inespecífica. El análisis de la información se realizó en el programa estadístico Stata versión 17 licenciado para la Pontificia Universidad Javeriana.

En cumplimiento de la Ley 1581 del 2012, se respeta la confidencialidad de los individuos y no se hará uso de datos que permitan su identificación. La base de datos se construyó de manera anónima, haciendo uso de la autorización de tratamiento de datos firmada en el HUSI. El uso de la información no generó cambios en la conducta tomada por el personal médico. El proyecto cuenta con la aprobación del Comité de Ética de Investigación de la Pontificia Universidad Javeriana.

## RESULTADOS

De los 1 119 pacientes diagnosticados con conjuntivitis infecciosa en nuestra institución en los periodos estudiados, 629 cumplieron con todos los criterios de inclusión del estudio. La edad promedio fue de 40,4 años. Se encontró que un poco más de la mitad eran mujeres (57,4%) (Tabla 1).

**Tabla 1:** Distribución de casos de conjuntivitis en relación con grupos de edad

Grupos de edad	Casos de conjuntivitis	%
0-18 años	63	10
19-40 años	294	46,7
41-94 años	272	43,3
Total	629	100

En los pacientes diagnosticados con conjuntivitis estudiados, 303 (48,2%) refirieron comorbilidades (Tabla 2). De estos, el 13,5% presentó cierto grado de inmunocompromiso (diabetes, inmunocompromiso y patología oncológica). Si bien no fue la comorbilidad más presentada en los pacientes que consultaron, se considera que son entidades que podrían favorecer y predisponer a las infecciones.

**Tabla 2:** Conjuntivitis y presentación de comorbilidades

Enfermedades	Número de pacientes	%
Diabetes mellitus	29	9,57
Inmunocompromiso	8	2,64
Patología oncológica	4	1,32
Hipertensión arterial	79	26,07
Hipotiroidismo	50	16,5
Patología ocular (degeneración macular, glaucoma, ojo seco, dacrioestenosis, ceguera, úlcera corneal, queratocono)	11	3,6
Patología respiratoria (asma, epoc, rinitis alérgica, bronquitis alérgica)	28	9,24
Otras patologías	94	31,02
Total	303	100

La frecuencia de presentación de los síntomas informados por los pacientes al momento del diagnóstico fueron la presencia de ojo rojo (81,7%), seguido de secreción (67,6%), prurito (36,9 %), ardor (34,3%), dolor ocular (30,5%), lagrimeo (27,8%), sensación de cuerpo extraño (2,1%), disminución de la agudeza visual (16,2%) y fotofobia (8,7 %). El 51,66 % de los casos se presentaron unilaterales en el momento del diagnóstico.

Según el tipo de clasificación identificada en las historias clínicas (Tabla 3) se encontraron: conjuntivitis viral, conjuntivitis viral sobreinfectada, bacteriana e inespecífica. La mayoría los pacientes presentaron conjuntivitis viral, seguida por conjuntivitis viral sobreinfectada, y en el 15,6 % de los casos consultaron inicialmente con conjuntivitis bacteriana. No hubo diferencias en el promedio de edad entre los diferentes tipos de conjuntivitis.

**Tabla 3:** Clasificación conjuntivitis

Tipo de conjuntivitis	Número de pacientes diagnosticados	%
Conjuntivitis viral	320	50,87
Conjuntivitis viral sobreinfectada	162	25,75
Conjuntivitis bacteriana	98	15,58
Conjuntivitis inespecífica	49	7,79

En cuanto al tratamiento reportado, se evidenció que el 28,8% (181) de los pacientes se automedicó antes de consultar a urgencias. El medicamento referido con mayor frecuencia fue el antibiótico combinado (38,1%), seguido de antibiótico solo (30,9%), lubricantes (16,6%), corticoides (3,3%), hipotensor ocular (2,8%), antialérgico (1,1%), antiviral (0,6 %) y otro tipo de medicamentos (6,6%).

De los 629 pacientes que hicieron parte del estudio, solo 33 eran usuarios de lentes de contacto, y de estos la mayoría (54,6%) presentó conjuntivitis viral, seguida del 24,2 % con conjuntivitis viral sobreinfectada.

En el periodo entre marzo del 2019 y marzo del 2021 (24 meses), se tuvo un total de 629 consultas. Entre marzo del 2019 y abril del 2020 se registraron 534 consultas al servicio de urgencias de la Unidad de Oftalmología, y a partir del día 24 de abril del 2020 (cuando entró en vigencia la Resolución 666 sobre medidas de bioseguridad) hasta marzo del 2021 se registraron 95 consultas.

**Figura 1:**
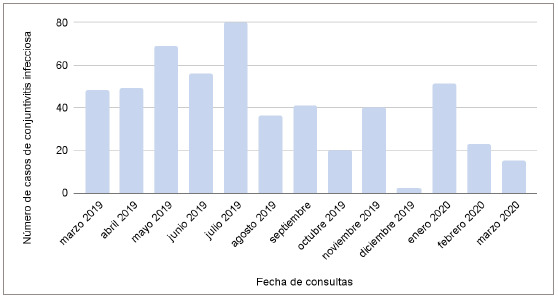
Casos de conjuntivitis infecciosa por fecha de consulta

En la Figura 1 se puede evidenciar que el mayor número de consultas fue en el mes de julio del 2019 (80 consultas), mientras que el menor número se dio en los meses de diciembre del 2019 y junio del 2020 (dos consultas en fecha).

En la Tabla 4 se recopilaron las presentaciones de conjuntivitis por periodos de tiempo. Se evidenció que la conjuntivitis viral seguida de la conjuntivitis viral sobreinfectada fueron las de mayor presentación durante el periodo de marzo del 2019 a diciembre del mismo año. En el año 2020 se evidencia una disminución del número de casos a menos de la mitad del registrado en el año anterior, y se mantiene la misma distribución de mayor frecuencia de presentación de conjuntivitis viral y viral sobreinfectada, como en el año 2019. Al observar los valores de presentación del año 2021, es evidente la reducción del número de casos de conjuntivitis infecciosa comparado con el año 2019.

**Tabla 4:** Tipos de conjuntivitis por periodos de tiempo

	Marzo 2019-Diciembre 31 2019	1 Enero 202031 diciembre 2020	Valor de P	Enero 2021-Marzo 2021	Valor de p
Conjuntivitis bacteriana	60	33	0,092799934	6	0,041638996
Conjuntivitis inespecífica	21	22		3	
Conjuntivitis viral	247	54		19	
Conjuntivitis viral sobreinfectada	113	39		12	
Total	441	148		40	

## DISCUSIÓN

Los hallazgos hechos en este estudio concuerdan con los reportados por Hashmi *et al.,* donde se evidencia que la mayor frecuencia de presentación de la conjuntivitis infecciosa se da entre los 20 y los 30 años, con mayor incidencia en las mujeres 8. Este resultado puede ser atribuible a que las mujeres tienden a consultar a los servicios de salud más que los hombres 9,10.

La literatura existente ha reportado una mayor incidencia de conjuntivitis en niños 8, no obstante, los resultados en la población objeto de estudio no muestran que haya un pico significativo de conjuntivitis en la infancia y la adolescencia.

Por otro lado, no se encontró ninguna asociación significativa entre la aparición de la conjuntivitis infecciosa y la patología de base de los pacientes. De igual manera, se esperaba 

que una mayor parte de la población fueran pacientes con diagnóstico de inmunosupresión, ya que este estado puede predisponer a la aparición de infecciones.

El diagnóstico de conjuntivitis infecciosa se basó en la clínica, por ende, no se tomaron muestras microbiológicas para la determinación del agente causal.

Durante la investigación se observó que la conjuntivitis viral fue la reportada con mayor frecuencia, sin embargo, se demuestra una disminución muy marcada de casos desde el inicio del confinamiento hasta el final del periodo de estudio.

Es necesario destacar que los virus que causan esta enfermedad con frecuencia, como los adenovirus, tienen una vía de transmisión similar a la del SARS-Cov-2 y es probable que las medidas tomadas durante el confinamiento hayan sido importantes para la disminución de casos en los meses siguientes a las directrices nacionales anticontagio 11.

Se mostró una menor cantidad de diagnósticos de todos los tipos de conjuntivitis evaluadas, por ende, se sugiere la hipótesis de que las medidas de cuidado, lavado de manos y aislamiento son eficaces para la reducción de contagios. Esta hipótesis fue observada en que hubo una reducción drástica del número de consultas por conjuntivitis en el periodo estudiado, con independencia de la edad y el sexo del paciente. El resultado más llamativo que emerge de los datos es que la cantidad de consultas se redujo significativamente después de marzo del 2020, momento en el que se estableció el confinamiento. Esta disminución es estadísticamente significativa y podría indicar así mismo la efectividad de las medidas de bioseguridad.

Se mostró también una menor cantidad de diagnósticos de todos los tipos de conjuntivitis evaluadas, lo que podría indicar que las medidas de cuidado, lavado de manos y aislamiento son eficaces para la reducción de contagios.

Los investigadores tienen la hipótesis de que la reducción importante en el número de consultas por conjuntivitis en el periodo estudiado, identificada después del mes de marzo del 2020, momento en el que se estableció el confinamiento, pudo deberse a la efectividad de las medidas de bioseguridad 12.

No obstante, la generalización de estos resultados está sujeta a ciertos interrogantes como, por ejemplo, la no asistencia al servicio de urgencias como reflejo de la situación de incertidumbre que se vivió a nivel mundial durante la pandemia 13-16. Así mismo, al tener más barreras es probable que pacientes de escasos recursos o de zonas rurales tuvieran más dificultades para el cuidado de su salud y el acceso a servicios de urgencias 17.

Además, es necesario destacar que espacios como colegios, centros comerciales y otros lugares de reunión, en los cuales se propagan con mayor facilidad los patógenos causantes de la conjuntivitis infecciosa, se encontraban cerrados.

Podemos concluir, a partir de los datos recolectados, que se pudo llegar a caracterizar a la población que consultó en el Hospital Universitario San Ignacio durante el periodo establecido del estudio.

Se hipotetizó que las medidas de bioseguridad como el aislamiento, el lavado frecuente de manos o el uso de tapabocas podían explicar la notoria reducción de los casos de propagación de conjuntivitis infecciosa en la comunidad. Estos datos son consistentes con los encontrados en la literatura internacional 18 ♦

## References

[1] Esteva Espinosa E (200). Conjuntivitis. Offarm.

[2] Azari AA, Barney NP (2013). Conjunctivitis: A systematic review of diagnosis and treatment. JAMA.

[3] Yeu E, Hauswirth S (2020). A Review of the differential diagnosis of acute infectious conjunctivitis: implications for treatment and management. Clin Ophthalmol.

[4] Rietveld RP, van Weert H, ter Riet G, Bindels P (2003). Diagnostic impact of signs and symptoms in acute infectious conjunctivitis: systematic literature search. BMJ.

[5] Eduardo N, Pinilla V, Martín-Gil I (2008). Conjuntivitis en el niño. FMC- Form Med Contin Aten Primaria.

[6] Alfonso S, Fawley J, Lu X (2015). Conjunctivitis. Clin Off Pract.

[7] Centro para el control y la prevención de enfermedades (US), Centro Nacional de Inmunización y Enfermedades Respiratorias (NCIRD), División de Enfermedades Virales (2019). Conjuntivitis.

[8] Hashmi MF, Gurnani B, Benson S (2022). Conjunctivitis.

[9] Keil J, Brendler V, Sachse C, Zülke A, Zeynalova S, Engel C, Loeffler M, Riedel-Heller SG, Konig HH, Stengler K (2020). Gender-specific differences in the utilization of health care services in an urban population sample. Gesundheitswesen.

[10] Redondo-Sendino A, Guallar-Castillón P, Benegas JR, Rodríguez-Artalejo F (2006). Gender differences in the utilization of health-care services among the older adult population of Spain. BMC Public Health.

[11] Ataee RA, Ataee MH, Mehrabi Tavana A, Salesi M (2017). Bacteriological aspects of hand washing: a key for health promotion and infections control. Int J Prev Med.

[12] Brueggemann AB, Jansen van Rensburg MJ, Shaw D, McCarthy ND, Jolley KA, Maiden MCJ (2021). Changes in the incidence of invasive disease due to Streptococcus pneumoniae, Haemophilus influenzae, and Neisseria meningitidis during the COVID-19 pandemic in 26 countries and territories in the Invasive Respiratory Infection Surveillance Initiative: a prospective analysis of surveillance data. Lancet.

[13] Garrafa E, Levaggi R, Miniaci R, Paolillo C (2020). When fear backfires: Emergency department accesses during the Covid-19 pandemic. Health Policy.

[14] Czeisler MÉ, Marynak K, Clarke KE, Salah Z, Shakya I, Thierry JM (2020). Delay or avoidance of medical care because of COVID-19-related concerns - United States, June 2020. MMWR Morb Mortal Wkly Rep.

[16] Boserup B, McKenney M, Elkbuli A (2020). The impact of the COVID-19 pandemic on emergency department visits and patient safety in the United States. Am J Emerg Med.

[16] Sürme Y, Ózmen N, Ertürk Arik B (2021). Fear of COVID-19 and related factors in emergency department patients. Int J Ment Health Addict.

[17] Peters HD, Garg A, Bloom G, Walker DG, Brieger WR, Rahman MH (2008). Poverty and access to health care in developing countries. Ann NY Acad Sci.

[18] Bachiller YC, Puente BG, Ibáñez LG, Benito GE, Duran MA, Dabad Moreno JV (2022). Pandemia COVID-19: impacto sobre la tasa de conjuntivitis virales. Arch Soc Esp Oftalmol.

